# College and Hospital Notes

**Published:** 1907-06

**Authors:** 


### COLLEGE AND HOSPITAL NOTES.

The Fordham Hospital at Pelham road and Southern boulevard, Borough of the Bronx, New York, recently dedicated its new buildings.

One is a five story structure and faces Bronx Park, in which are six wards. The first floor is devoted to children. The nurses' home is in the rear of the hospital.

Dr. John W. Brannan, president of the board of trustees, was master of ceremonies. Brief addresses were made by Dr. Thomas Darlington, president of the Department of Health; the Rev. Daniel Quinn, president of Fordham University: Patrick F. McGowan, president of the Board of Aldermen, and Justice John M. Tierney, of the Municipal Court.

---

A Summer School will be held at Syracuse University for six weeks, July 5 to August 16, 1907. This school is designed to accommodate (1) teachers of elementary and secondary schools; (2) students who may desire to review college preparatory work; or (3) to prepare for state preliminary examinations; (4) students desiring to do college work during the summer. Credit will be given for work under (4).

Libraries and laboratories will be open and available. Tuition for two or more courses, $25, for one course, $15. Thoroughly competent men will give the instruction. Courses will be offered in Greek, Latin, German, French, Spanish, Public Speaking, Philosophy, English, History, Political Economy, Sociology, Mathematics, Physics, Chemistry, Biology, and in other subjects if arranged for in advance. For circular and information address The Registrar, Syracuse University.

---

The Jackson Health Resort training school at Dansville, held its graduation exercises for nurses May 14, 1907. Nine nurses graduated and the address to the class was delivered by Dr. William P. Spratling, superintendent of the Craig Colony at Sonyea.

---

An Emergency Hospital was established at the Actors' Fair, recently held at the Metropolitan Opera House, New York. The hospital registry recorded nearly 200 patients cared for during the fair.

The idea was conceived by Mrs. A. M. Palmer, one of the managers of the fair, and carried out by Dr. Jessie T. Bogle, who had charge of the undertaking.

Dr. Bogle was assisted by an able corps of physicians, one or more of whom were in constant attendance during the progress of the fair, and also by the Post-Graduate Hospital, the latter having rendered invaluable aid by furnishing the ward in the most approved manner, and also the nurses, three of whom were always on duty.

The hospital was fully equipped with everything that would possibly be needed in treating cases of sudden illness or accidents of every kind.

---

The Alumni Association, of the Medical Department, University of Buffalo, will hold its thirty-second annual meeting May 28, 29, 30 and 31, 1907. Although this issue of the Journal will hardly reach many of the alumni before the meeting is well under way, we yet publish this official program to give it permanent record, as a tribute to the old University, to the executive committee and to all who have contributed to make commencement week so interesting in celebration of the sixty-second anniversary of the grand old college.

Tuesday, May 28.

8.00 P.M.—The exercises will begin with a reception in the college building, by the faculty of the department, and the association will transact routine business and elect officers, and the classes '47, '57, '67, '77, '82, '87 and '97 will be received by the faculty and officers of the association.

9.00 P.M.—The executive committee is pleased to announce that Honorable Charles P. Norton, Vice Chancellor of the University of Buffalo has consented to tell the association why Buffalo is destined to be a university center.

10.00 P.M.—A luncheon will be spread in the library of the college.

Wednesday, May 29.

10.00 A.M.—The clinics will begin at the Buffalo General Hospital.

Dr. Matthew D. Mann, Dean and Professor of Obstetrics and Gynecology, will give a clinic in Diseases of Women.

11.00 A.M.—Dr. Roswell Park, Professor of Principles and Practice of Surgery and Clinical Surgery, will give a clinic in General Surgery.

12.30 P.M.—The trustees of the Buffalo General Hospital, have very generously provided a luncheon at the hospital for the alumni of the medical department of the University of Buffalo.

1.30 P.M.—Dr. Charles G. Stockton, Professor of Principles and Practice of Medicine and Clinical Medicine, will give a clinic in Internal Medicine.

3:00 P.M.—Dr. Ernest Wende, Health Commissioner of Buffalo, and Professor of Dermatology, will give a clinic in Diseases of the Skin.

3.30 P.M.—Dr. James W. Putnam, Professor of Diseases of the Nervous System will give a clinic in Neurology.

3.45 P.M.—Dr. William C. Krauss, will give a clinic in Diseases of the Mind.

4.00 P.M. to 6.00 P.M.—Dr. Charles Cary '75, will receive at his home, 340 Delaware Avenue, in the afternoon of Wednesday, May twenty-ninth, from four to six. Through the executive committee each alumnus of the medical department of the University of Buffalo, is invited.

8.00 P.M.—Wednesday evening, May 29th, has been set aside as Fraternity Night. The "I. C. I.," the "A. O. D.," the "O. U. P.," and the "N. S. N.," have each prepared a program for the entertainment of its alumni, for which each has issued invitations. It is hoped that the alumni will enjoy the hospitality of the underclassmen.

THURSDAY, MAY 30.

10.00 A.M.—The clinics continue at the Buffalo General Hospital, Dr. Charles Cary, Professor of Clinical Medicine will give a clinic in Internal Medicine.

11.00 A.M.—Dr. Irving M. Snow, Clinical Professor of Pediatrics, in conjunction with Dr. DeWitt H. Sherman, Adjunct Professor of Therapeutics, will give clinics in Diseases of Children.

11.30 A.M.—Dr. William C. Phelps, will give a clinic in General Surgery. Dr. John Parmenter, Professor of Clinical Surgery, will give a clinic in General Surgery.

1.00 P.M.—The association will give a luncheon at the Saturn Club, corner Delaware avenue and Edward street, to its members.

3.00 P.M.—The clinics will continue at the Hospital of the Sisters of Charity, Main street. Dr. Henry C. Buswell, Adjunct Professor of Clinical Medicine, will give a clinic in Internal Medicine.

4.00 P.M.—Dr. Edward J. Meyer, Adjunct Professor of Clinical Surgery, will give a clinic in General Surgery.

8.30 P.M.—Thursday evening, May 30, has been set aside as Free-Lance Night.

The Buffalo Academy of Medicine will have a meeting to which the alumni are especially invited.

Class dinners, smokers and theatre parties will occur as determined.

The executive committee has arranged to provide seats for the various performances at the theatres. Tickets for choice locations will be on sale at the treasurer's desk until 1 P.M., Friday, May 31.

FRIDAY, MAY 31.

11.00 A.M.—The Sixty-second Annual Commencement Exercises of the University of Buffalo, including the Departments of Medicine, Pharmacy, Dentistry and Law will take place at the Teck theatre, Main and Edward Streets.

Jeremiah Whipple Jenks, A.M., Ph. L.L.D., Professor of Political Economy and Politics, Cornell University, will deliver the address.

1.00 P.M.—The faculty of the Medical Department will receive the graduates, their friends and the alumni at the University Club, corner Delaware avenue and Allen street, where a luncheon will be served.

SPECIAL CLINICS.

The executive committee is pleased to announce that the following gentlemen will give clinics in the special subjects set down. The time and place to be announced upon the bulletin board in the College Hall.

EYE.—Dr. Lucien Howe, Clinical Professor of Ophthalmology; Dr. Arthur G. Bennett, Instructor in Ophthalmology; Dr. John D. Flagg, Instructor in Ophthalmology.

EAR.—Dr. George F. Çott, Clinical Professor of Otology.

NOSE.—Dr. Frank W. Hinkel, Clinical Professor of Laryngology; Dr. W. Scott Renner, Clinical Professor of Laryngology; Dr. James J. Mooney, Clinical Lecturer in Laryngology; Dr. Henry J. Mulford, Lecturer in Laryngology.

GYNECOLOGY.—Dr. Lawrence H. Hanley, Clinical Professor of Obstetrics.

SKIN.—Dr. Alfred E. Diehl, Adjunct Clinical Professor of Dermatology; Dr. Grover W. Wende, Clinical Professor of Dermatology.

